# Design, *in silico* and pharmacological evaluation of a peptide inhibitor of BACE-1

**DOI:** 10.3389/fphar.2023.1184006

**Published:** 2023-06-16

**Authors:** Renata Boldin, Bianca Cestari Zychar, Luis Roberto C. Gonçalves, Juliana Mozer Sciani

**Affiliations:** ^1^ Unidade de Farmacologia e Gastroenterologia (UNIFAG), Universidade São Francisco, Bragança Paulista, São Paulo, Brazil; ^2^ Laboratório de Farmacologia Molecular e Compostos Bioativos, Universidade São Francisco, Bragança Paulista, São Paulo, Brazil; ^3^ Laboratório de Fisiopatologia, Instituto Butantan, São Paulo, Brazil

**Keywords:** peptide, BACE-1, competitive inhibitor, *in silico* design, pharmacokinetics

## Abstract

**Introduction:** Alzheimer’s disease (AD) is the main type of dementia, caused by the accumulation of amyloid plaques, formed by amyloid peptides after being processed from amyloid precursor protein (APP) by *γ*- and *ß*-secretases (BACE-1). Although amyloid peptides have been well established for AD, they have been found in other neurodegenerative diseases, such as Parkinson’s disease, Lewy body dementia, and amyotrophic lateral sclerosis. Inhibitors of BACE-1 have been searched and developed, but clinical trials failed due to lack of efficacy or toxicity. Nevertheless, it is still considered a good therapeutic target, as it was proven to remove amyloid peptides and improve memory.

**Methods:** In this work, we designed a peptide based on a sequence obtained from the marine fish Merluccius productus and evaluated it by molecular docking to verify its binding to BACE-1, which was tested experimentally by enzymatic kinetics and cell culture assays. The peptide was injected in healthy mice to study its pharmacokinetics and toxicity.

**Results:** We could obtain a new sequence in which the first N-terminal amino acids and the last one bound to the catalytic site of BACE-1 and showed high stability and hydrophobicity. The synthetic peptide showed a competitive inhibition of BACE-1 and Ki = 94 nM, and when injected in differentiated neurons, it could reduce Aβ42o production. In plasma, its half-life is ∼1 h, clearance is 0.0015 μg/L/h, and Vss is 0.0015 μg/L/h. The peptide was found in the spleen and liver 30 min after injection and reduced its level after that, when it was quantified in the kidneys, indicating its fast distribution and urinary excretion. Interestingly, the peptide was found in the brain 2 h after its administration. Histological analysis showed no morphological alteration in any organ, as well as the absence of inflammatory cells, indicating a lack of toxicity.

**Discussion:** We obtained a new BACE-1 inhibitor peptide with fast distribution to the tissues, without accumulation in any organ, but found in the brain, with the possibility to reach its molecular target, BACE-1, contributing to the reduction in the amyloid peptide, which causes amyloid-linked neurodegenerative diseases.

## 1 Introduction

*β*-Secretase-1 (BACE-1) is an aspartic-like type 1 transmembrane peptidase belonging to the pepsin family. This enzyme contains two aspartic acid residues in its active site (Asp32/Asp228) (Rawlings et al., 2018) and a flexible flap in the region of amino acid positions 67 to 77, which controls substrate binding (Xu et al., 2012).

BACE-1 has the substrate amyloid precursor protein (APP) and generates amyloid plaques in the brain, causing several amyloid-linked neurodegenerative diseases. BACE-2 is 64% similar to BACE-1 but is not related to amyloid peptide formation in the brain (Yan and Vassar, 2014).

Alzheimer’s disease (AD), the main type of dementia, is characterized by memory loss and cognitive impairment. It is common in the elderly, and due to increased life expectancy, the number of cases increased to 46.8 million people in 2015, and it is now the seventh leading cause of death in the world. The expectation is that, in 2050, there will be 131.5 million cases (Alzheimer’s Disease International, 2022).

This disease is caused by the formation of amyloid plaques and neurofibrillary tangles, which induce dystrophic neurites and significant neuronal loss in specific regions of the brain (and consequently, loss of synaptic contact), in addition to chronic inflammation and oxidative damage (Hardy and Selkoe, 2002).

Amyloid plaques are formed initially by APP or amyloid *ß* precursor protein processing, after cleavage of the C-terminal portion by *a*-secretases, *ß*-secretases, and *γ*-secretases (Wakabayashi and De Strooper, 2008).

In the first pathway, named the non-amyloidogenic pathway, APP is cleaved by *a*-secretase, producing sAPPalpha, which is excreted into the extracellular medium, in addition to a peptide, generated after *γ*-secretase cleavage. In the second pathway, referred to as the amyloidogenic pathway, APP is first cleaved by *ß*-secretase producing a fragment of 99 amino acids. Another enzyme, *γ*-secretase, performs a new process to release the amyloid peptide. The last cleavage occurs non-specifically, and a peptide of 40 or 42 amino acids (Aβ40 and Aβ42) can be generated, which is toxic to neurons (Murphy and LeVine, 2010; Soria Lopez et al., 2019).

Aβ40 and Aβ42 oligomerize in the aqueous environment of the brain. These aggregated insoluble peptides, known as amyloid plaques, accumulate in specific extracellular regions of the brain, inside or outside the cells, and cause AD (Hardy and Selkoe, 2002). Although amyloid peptides have been well established for AD, they have been found in other neurodegenerative diseases, such as Parkinson’s disease, Lewy body dementia, and amyotrophic lateral sclerosis (Calingasan et al., 2005; Lim et al., 2019; Biundo et al., 2021).

Several studies have searched for a BACE-1 inhibitor to control neurodegenerative diseases, especially AD. However, clinical trials have failed due to lack of efficacy or toxicity (Das and Yan, 2019). Nevertheless, the beneficial effects of enzyme inhibition have been demonstrated, including the reduction of Aβ levels and improvement of memory (Vassar, 2014).

Currently, only two pharmacological groups have been approved for the treatment of this disease, namely, acetylcholinesterase inhibitors and N-methyl-D-aspartate glutamatergic receptor antagonists. Few drugs licensed by regulatory agencies, such as tacrine, rivastigmine, donepezil, and galantamine, are commercially available for the treatment of AD. However, these drugs only increase patients’ life expectancy by a few years and do not represent a cure (Hung and Fu, 2017).

Recently, a monoclonal antibody was approved by the FDA for AD treatment, as it was shown to be effective in removing amyloid plaques; however, several adverse effects have been reported. Nevertheless, these data are important because it was confirmed that the amyloid peptide is still a relevant pharmacological target (Shi et al., 2022).

Peptides have been studied to inhibit BACE-1, and they are biomolecules that fill the gap between small molecules and protein drugs and share the advantages of both groups, namely, high stability and bioavailability (Bruno et al., 2013; Craik et al., 2013). Animal venoms and secretions (including marine ones) are rich in bioactive peptides, representing a successful group of molecules with high potency that are rarely studied and explored from a pharmacological aspect (Bhat et al., 2015; Sciani et al., 2016).

Thus, the aim of this study was to obtain a new peptide derived from a natural one isolated from a marine fish, designed with the help of computational tools, and evaluate its inhibitory activity on BACE-1, in addition to its pharmacokinetic properties.

## 2 Materials and methods

### 2.1 Peptide

A peptide isolated from *Merluccius productus* fish protein hydrolysate (Lee et al., 2019) was modified by removing an Asp residue and searched in BLAST (Basic Local Alignment Search Tool) for similarity analysis, considering a non-redundant database, without organism selection, matrix BLOSUM62, and gap existence. Moreover, the new peptide was analyzed as its physico-chemical proprieties using the ProtParam Tool (ExPASy), in which molecular mass, pI, instability index, GRAVY (grand average of the hydropathicity index), and aliphatic index were calculated (Gasteiger et al., 2005).

### 2.2 Molecular docking

To evaluate the potential of the new peptide to inhibit BACE-1, we first performed molecular docking experiments. The target protein 2VKM was selected from the PDB (Protein Data Bank) based on the resolution by the X-ray diffraction method (∼2 Å), isolated from humans, and had its experimental structure determined together with an inhibitor to serve as an anchorage model. Other BACE-1 with different flap positions were also studied—PDB code 1W50, 2QU3, 2VIE, 3EXO, and 3HVG.

The protein file (PDB) was prepared using UCSF Chimera v1.15 and AutoDockTools (version 1.5.6) to remove the native ligand, missing atoms, chain breaks, and water molecules. Hydrogens were added at a pH of 7.0, and charges were added to Asp32 and Asp228 or both. The grid was centered within the protein’s active site with the size of 22 × 24 × 28 Å in the *x*, *y*, and *z* axes, respectively, and positions 1.223 for *x*, −1.18 for *y*, and 37.361 for *z*.

The peptide sequence was inserted using the MDockPeP server (Yan et al., 2016; Xu et al., 2018) in the FASTA format. The cutoff of bRMSD for the restriction of the peptide conformation was set at 5.5 Å, and the exhaustiveness value for the sampling was 100. Analysis of the peptide position into BACE-1, amino acid bind determination, and distances between atoms were performed using UCSF Chimera v1.15.

### 2.3 ADME and toxicological analysis (*in silico*) of the peptide

Predictions were performed using the PeptideMass platform to check for possible points of processing of the peptide by plasma and tissue enzymes.

Half-life in mammals was estimated using the ProtParam Tool (ExPASy). To estimate toxicity in animals and cells, the peptide sequence was analyzed using the ToxinPred (Gupta et al., 2013). The tool predicts based on the observations of several motifs present in toxic peptides for humans, deposited in SwissProt, where they are used as a template for the prediction of toxic peptides, searched by the MEME software, and followed by the query of “toxic” sequences by the MAST software.

The structure of the peptide was estimated using the MDockPeP server and UCSF Chimera v1.15.

### 2.4 Enzymatic kinetics on BACE-1

The peptide was commercially obtained (manufactured by Biotik, commercialized by FastBio, Brazil) and synthetized by the solid phase with >95% purity. The peptide was tested using an enzymatic kinetics model, in which 10 μM of peptide (diluted in 0.9% saline solution) was incubated with recombinant BACE-1 and reaction buffer for 15 min at room temperature (MAK237, Sigma-Aldrich, St Louis, MO, United States). After that, H-Arg-Glu (EDANS)Glu-Val-Asn-Leu-Asp-Ala-Glu-Phe-Lys (DABCYL)Arg-OH quencher substrate (0–32 µM) was added to the reaction mixture. Readings were taken using a fluorimeter (Glomax, Promega, Madison, WI, United States) every 5 min at 37° in a λ_ex_ = 335 and λ_em_ = 510. Experiments were conducted in triplicate in two independent sets, and the mean of fluorescence was obtained for further calculations. The velocity of the reaction was calculated as ∆ fluorescence values at ∆ time for each substrate concentration. 1/V and 1/S values were obtained and plotted in a graph (Lineweaver–Burk plot), and kinetic parameters (slope, Vmax, Km, and Ki) were calculated as the mean ± SD using GraphPad Prism v5.

### 2.5 Pharmacokinetics

#### 2.5.1 Animal injection and sample preparation

Authorization for pharmacokinetics analyses was obtained from the Ethics Committee of Instituto Butantan (CEUAIB 58872400322), where the experiments with animals were carried out. Healthy adult male Swiss mice weighing approximately 25 g were used. All animals were placed in microisolators for a 12-h light/dark cycle, 70% humidity, and a constant average temperature (22°C).

A single dose of the peptide diluted in sterile PBS (pH 7.4) was injected intravenously in the bolus into the caudal vein of mice (*n* = 3) at a concentration of 4 mg/kg (0.2 mL). A group of animals (*n* = 3) received the sterile saline solution for the plasma and organ control, without the peptide (named ‘basal’). Blood was taken after 0, 5, and 30 min and 2, 6, and 24 h after injection. At these times, blood was collected via the retro-orbital route using a Pasteur pipette and dispensed into plastic tubes containing EDTA. The final blood volume was subjected to centrifugation at 1,500 rpm for 10 min at room temperature to obtain plasma, which was stored in a −80°C freezer until the analysis.

Moreover, organs were obtained at the same times, after a lethal injection of anesthetics (ketamine 300 mg/kg and xylazine 30 mg/kg). The organs collected included the brain, pancreas, spleen, kidneys, lungs, and liver. The organs were washed with the saline solution to remove excess blood, frozen in liquid nitrogen, and kept at −80°C until use. For the experiments, organs were submerged in 1 mL of 50 mM PBS with pH 7.5 containing peptidases inhibitors (aprotinin, bestatin, E-64, leupeptin, and pepstatin A, a protease inhibitor cocktail, Sigma-Aldrich) and lysed in a tissue and cell disruptor (ULTRA-TURRAX IKA, Staufen, Germany). The lysate was centrifuged for 2 min at 13,200 rpm at 4°C, and the supernatant resulting from centrifugation was stored in a −80°C freezer until the analysis.

Both plasma and organs (150 μL) were incubated with 100 mM DL-dithiothreitol at room temperature for 30 min; after that, they were incubated to 300 mM iodoacetamide at room temperature for another 30 min in the dark. Subsequently, 150 μL of 0.1% formic acid solution and 400 μL of Milli-Q water were added and agitated for 5 min. The samples were then submitted to solid-phase extraction using a C18 cartridge (Supelco) with elution in a 60:40 acetonitrile:water solution (v/v). The eluate was inserted by mass spectrometry for analysis and quantification.

The solid-phase extraction was performed with plasma added to a known concentration of the peptide to set the mass spectrometric conditions.

#### 2.5.2 Mass spectrometry and pharmacokinetics analysis

The determination and quantification of the peptide in plasma and organs of mice were performed by liquid chromatography (UPLC system, Waters Co.) coupled to mass spectrometry ESI-Xevo TQ-S (Waters Co., United States). The samples were inserted in a C18 column (Luna, 5 μm, 150 × 4.6 mm, Phenomenex) and eluted by 70% methanol in water, containing 0.05% formic acid in a constant flow of 0.3 mL/min. The ion was monitored at the positive ionization mode, 150°C source temperature, 550°C desolvation temperature, 800 L/h desolvation gas flow, 2 kV for capillary, and 20 kV cone voltage. Data were acquired and analyzed using MassLynx 4.

To define the relationship between the instrument response and the known concentration of the peptide, a calibration curve with six points of the peptide (0.1–20 μg/mL for plasma and 5–40 μg/mL for organs) was generated in a range defined according to the sensitivity of the method and the expected concentrations of the samples in animals (based on the amount of peptide injected).

Each analysis lot contained the following samples: a calibration curve consisting of a blank sample (peptide-free biological matrix), samples containing peptide standard, and plasma or organ samples. Calibration functions were calculated using the peak areas of the peptide. The peptide concentrations in the samples were calculated from the weighted linear regression equation (1/×2) obtained from the calibration curve (peptide concentration as the function of the area). This regression model (1/×2) was used because of the amplitude of the calibration curve.

The plasma concentration (Cp), in µg/L, was determined by mass spectrometric analysis, and then, area data were analyzed using the PKSolver add-in program for Microsoft Excel. Pharmacokinetics parameters as the natural logarithm of Cp (lnCp), Cp0, half-life, and clearance were calculated in a compartmental analysis of plasma data after the intravenous bolus input (Zhang et al., 2010). For organs, data are present as mean ± SD for each point.

### 2.6 Histology

Organs were removed and immediately fixed in Bouin’s solution for processing by dehydration ethanol sessions and historesin embedding to obtain 3 µm thick transversal slides. Sections were stained with hematoxylin–eosin and examined using a light microscope for a qualitative analysis.

### 2.7 BACE-1 activation

To verify if the peptide could inhibit BACE-1, we used cultured differentiated neurons and stimulated them to activate the enzyme and release amyloid peptide, detected by thioflavin-T labeling. SH-SY5Y cells (ECACC, Sigma-Aldrich, St. Louis, MO, United States), cultivated with Dulbecco’s modified Eagle’s medium/Nutrient Mixture F-12 (DMEM/F-12) (1:1) (Gibco Life Technologies, Grand Island, NY, United States) and 10% heat-inactivated fetal bovine serum (FBS), were adhered to a 96-well plate (1 × 10^3^ cells/well), then differentiated by replacing them to the media DMEM/F-12 supplemented with 2% FBS and 10 µM retinoid acid (Sigma-Aldrich, Saint Louis, MO), and maintained in a humidified atmosphere of 5% CO_2_ at 37°C. This media was replaced every 2 days, until the eighth day, when neurons were treated with 100 µM H_2_O_2_ for 6 h, in the absence or presence of peptide (10 µM). After that, the cell media was removed, and a 10 µL aliquot was added to 88 µL of phosphate-saline buffer (PBS 50 mM and pH 7.2) and 2 µL thioflavin of 1 mM. The solution was read using the fluorimeter in a λ_ex_ = 450 nm and λ_em_ = 490 nm. Means of arbitrary units of fluorescence (AUF) of three experiments were calculated along with SD, and a statistical analysis was applied using one-way ANOVA for a three-group comparison (control, H_2_O_2_, and H_2_O_2_ + peptide), followed by Tukey’s post-test using GraphPad Prism v5. Statistical significances were considered when *p* < 0.05.

## 3 Results

### 3.1 Peptide properties and BACE-1 docking

**FIGURE 1:**
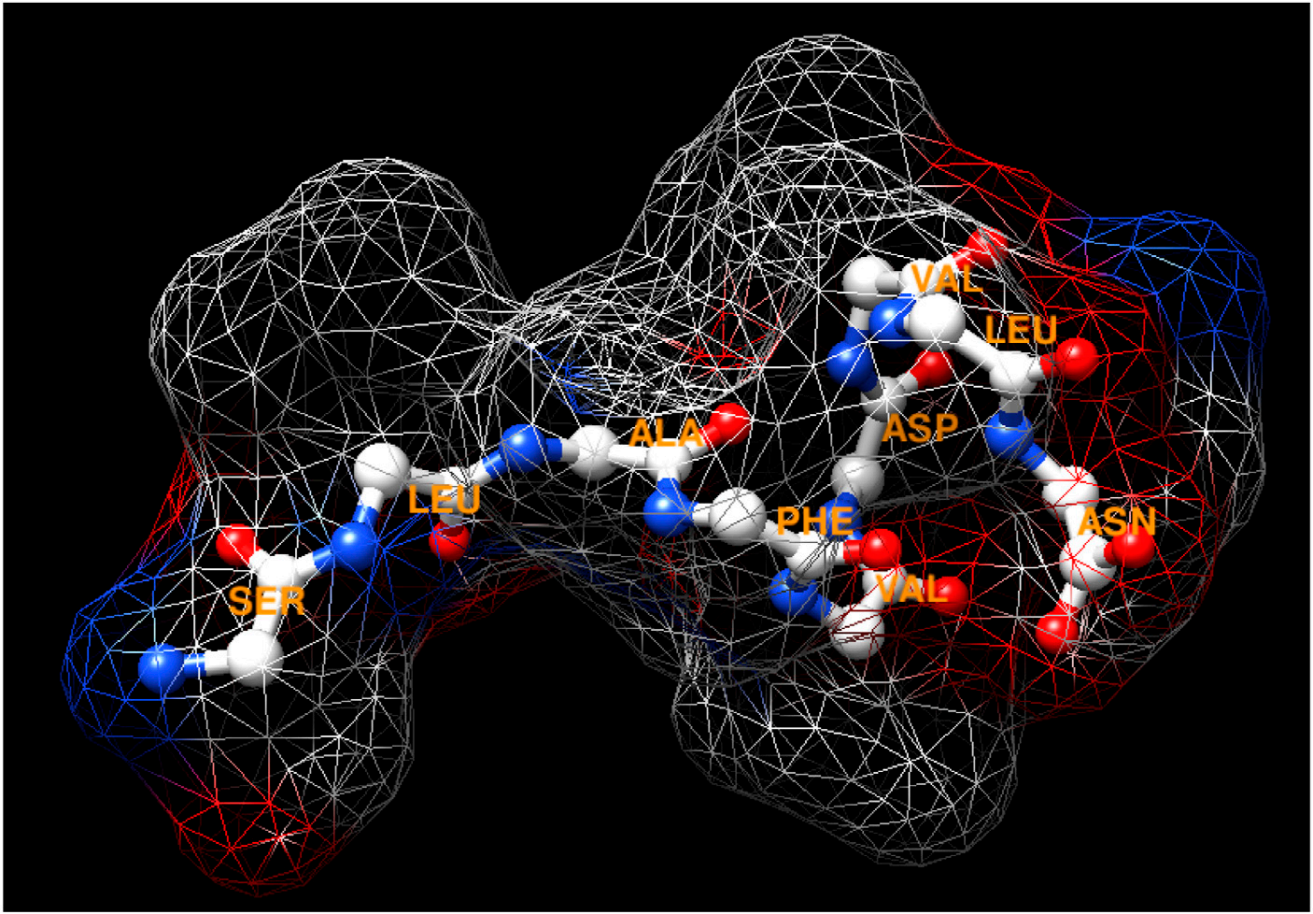
Tridimensional structure of the peptide by ribbon and surface, according to its amino acid side chains. The structure shows amino acids (three-letter), nitrogen in red, and oxygen in blue.

The peptide has the sequence SLAFVDVLN, a molecular mass determined as 977.73 by mass spectrometry, and its tridimensional structure is depicted in Figure 1. The amino acid sequence was not found in the database, indicating a new peptide. Its properties were estimated using *in silico* tools and are presented in Table 1. It can be observed that pI is acidic, indicating that the peptide is charged at neuronal pH. On the other hand, it is stable and has a high aliphatic index value, which reflects thermic stability. Moreover, the peptide contains six hydrophobic residues, reflecting high value of GRAVY (hydrophobicity), and also has one hydrophilic residue at both the N- and C-terminal, resulting in an amphipathic characteristic that is sufficient to permeate biological membranes. The presence of only one acid residue contributes to this membrane permeation.

**TABLE 1:** Properties of the peptide estimated computationally.

Molecular mass	pI	Instability index	GRAVY	Aliphatic index	Half-life (h)
977.13	3.80	−7.81 (stable)	1.42	162.22	1.90

Moreover, it has a half-life of 1.9 h and does not contain specific amino acids for cleavage by plasma enzymes.

The binding portion of the peptide to the catalytic site of BACE-1 was evaluated by molecular docking. The protein selected in PDB was the code 2VKM, with a resolution of 2.05 Å obtained by X-ray diffraction after expression in bacteria but with a human sequence.

**FIGURE 2:**
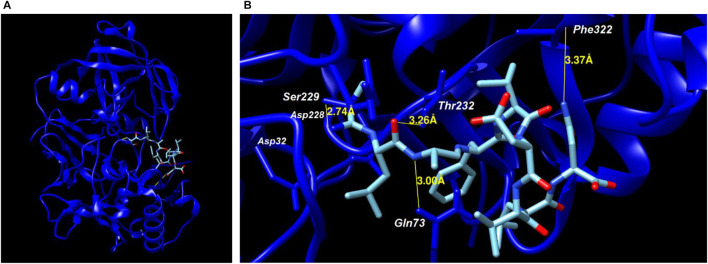
Molecular docking of BACE-1 (2VKM PDB code) and peptide. **(A)** Positioning of the peptide on the catalytic site of the enzyme. **(B)** Binding amino acids from the enzyme and distances to the peptide.

Four amino acids from the peptide bound to the side chains of amino acids from the active site of BACE-1, with a distance of up to 3.37 Å, being the first three (Ser–Leu–Ala) from the N-terminal portion and one (Asn) from the C-terminal (Figure 2). The binding profiles (amino acids, elements, and distances) are shown in Table 2, where it is possible to see the binding in the amino acid next to the catalytic site composed by Asp32/Asp228, besides the Gln73 from the flap. The ITScorePeP was calculated as −138.00. The distances to the amino acids Asp32 and Asp228 were 7.55 and 6.94 Å, respectively. The docking showed in Figure 2 was performed with protonated Asp228, but similar results in terms of bound amino acids were found when protonated Asp32 was analyzed.

**TABLE 2:** Protein–peptide binding profile and distance between amino acids.

Peptide amino acid	Protein amino acid	Distance (Å)
Ala3 (N)	Gln73	3.00
Leu2 (O)	Thr232	3.26
Ser1 (N)	Ser229	2.74
Asn9 (N)	Phe322	3.37

The peptide was also docked with BACE-1 in different flap positions (Xu et al., 2012)—PDB codes 1W50, 2QU3, 2VIE, 3EXO, and 3HVG. No change in binding was detected in terms of amino acid binding, but there were slight differences in the distances (data not shown). The lowest docking score was obtained with BACE with PDB code 3EXO (−129.20).

### 3.2 Enzymatic activity on BACE-1

**FIGURE 3:**
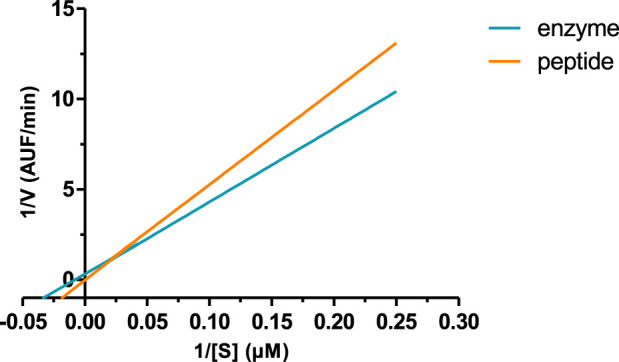
Lineweaver–Burk plot of BACE-1 activity and the enzyme in the presence of the peptide.

The peptide, when incubated with recombinant BACE-1, slightly reduced the velocity of the reaction compared to the enzyme without any treatment. Figure 3 shows the velocity of the reaction with or without the peptide in a Lineweaver–Burk plot, where it is possible to see differences in the slope and intersection of the *x* and *y* axes, reflecting different Vmax and Km values (shown in Table 3). This profile indicates that the peptide is a competitive inhibitor, or even a mixed inhibition, as Km value increased, and 1/V increased in the presence of the peptide.

**TABLE 3:** Enzymatic kinetics parameters were obtained for BACE-1 activity, and the enzyme was incubated with the peptide. Values are mean ± SD.

	BACE-1	BACE-1 + peptide
Vmax	0.2352 ± 0.03233	0.0503 ± 0.01044
Km (µM)	−0.0060	−0.09
Ki (µM)		0.0940

### 3.3 Pharmacokinetics

**FIGURE 4:**
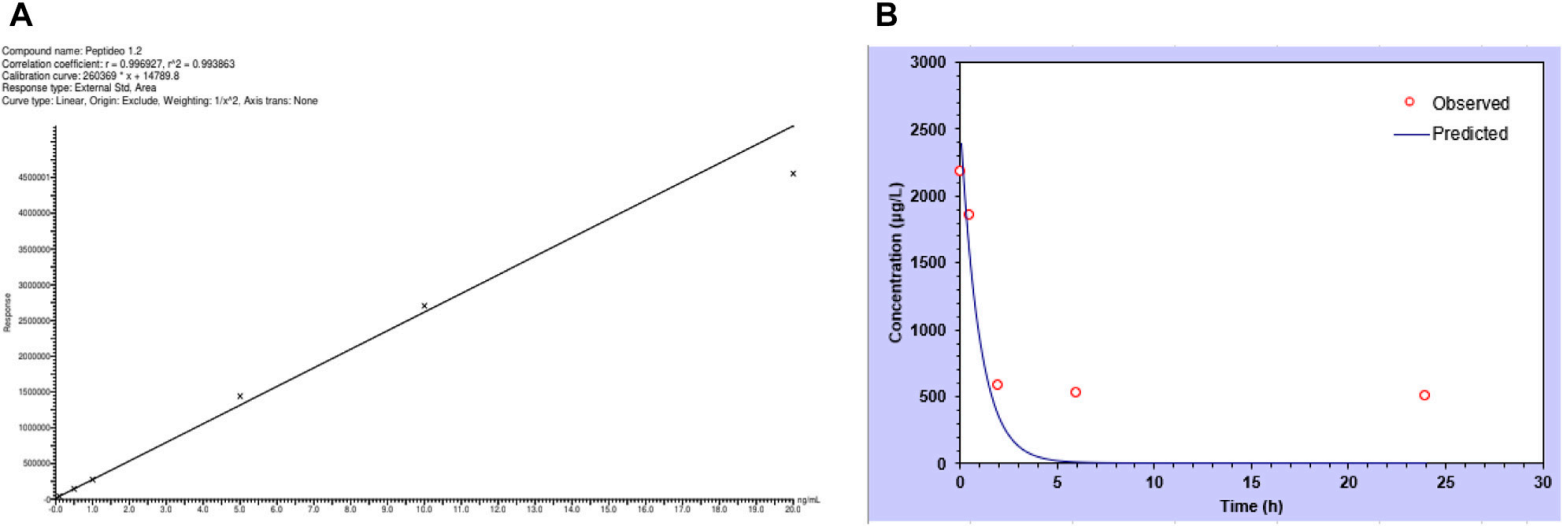
Peptide quantification in plasma. **(A)** Calibration curve of the peptide analyzed by mass spectrometry. Each point represents the area detected. **(B)** Concentration of the peptide in mouse plasma, with values observed experimentally and predicted for pharmacokinetics calculation parameters. Values represent the mean of three animals.

The calibration curve for the peptide in plasma resulted in a correlation coefficient greater than 0.99, indicating that the method was linear (Figure 4A). The LLOQ peak area was at least five times greater than interferences observed in the same retention-time peptide, when a blank sample was analyzed. The deviation was less than or equal to 20% in relation to the nominal concentration for the LLOQ and less than or equal to ±15% of the nominal concentration for the other concentrations in the calibration curve.

When the plasma and organs were analyzed using the same method, it was possible to quantify the peptide in such biological matrices. Figure 4B shows the plasma concentration of the peptide in the natural log over time. From these data, it was possible to calculate pharmacokinetic parameters, and values are presented in Table 4, where it is possible to see a short half-life (0.7 h) but an important volume of distribution and elimination.

**TABLE 4:** Pharmacokinetic parameters of the peptide quantified in mouse plasma.

Parameter	Unit	Value
t1/2	h	0.69
Cp0	μg/L	2,595.96
V	(mg/kg)/(μg/L)	0.00154
CL	(mg/kg)/(μg/L)/h	0.00154
AUC 0-t	μg/L*h	2,595.96
AUC 0-inf	μg/L*h	2,595.96
AUMC	μg/L*h^2	2,595.96
MRT	h	1
Vss	mg/kg/(μg/L)	0.00150

**FIGURE 5:**
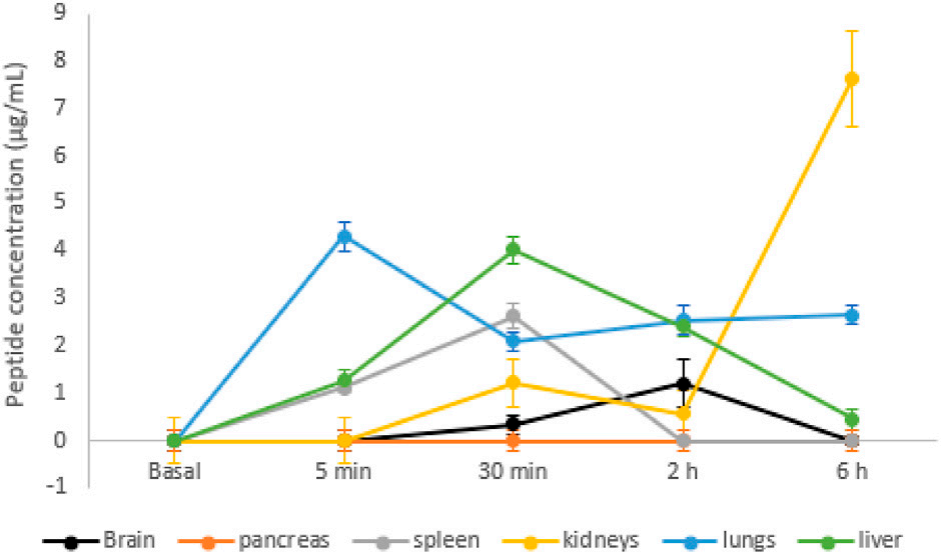
Peptide detected in mouse organs by mass spectrometry over time. Data are shown as mean ± SD (*n* = 3).

Organs were also evaluated using mass spectrometry to quantify the peptide present each time. As shown in Figure 5, no signal was detected in the animals that did not receive the peptide. Five minutes after peptide injection, it was possible to observe the peptide in the spleen, liver, and lungs.

In the spleen and liver, the concentration increased for up to 30 min after injection and decreased over time, with the liver showing slower elimination. It was not possible to identify the peptide in the pancreas. In the kidneys, the concentration of the peptide increased 30 min after the injection, slightly decreased after 2 h, but increased after 6 h after the injection. Five minutes after the injection, a high concentration of the peptide was found in the lungs, with levels reduced and maintained constant for up to 6 h. In the brain, the peptide reached a 2-h peak after injection and decreased after 6 h.

### 3.4 Histology

**FIGURE 6:**
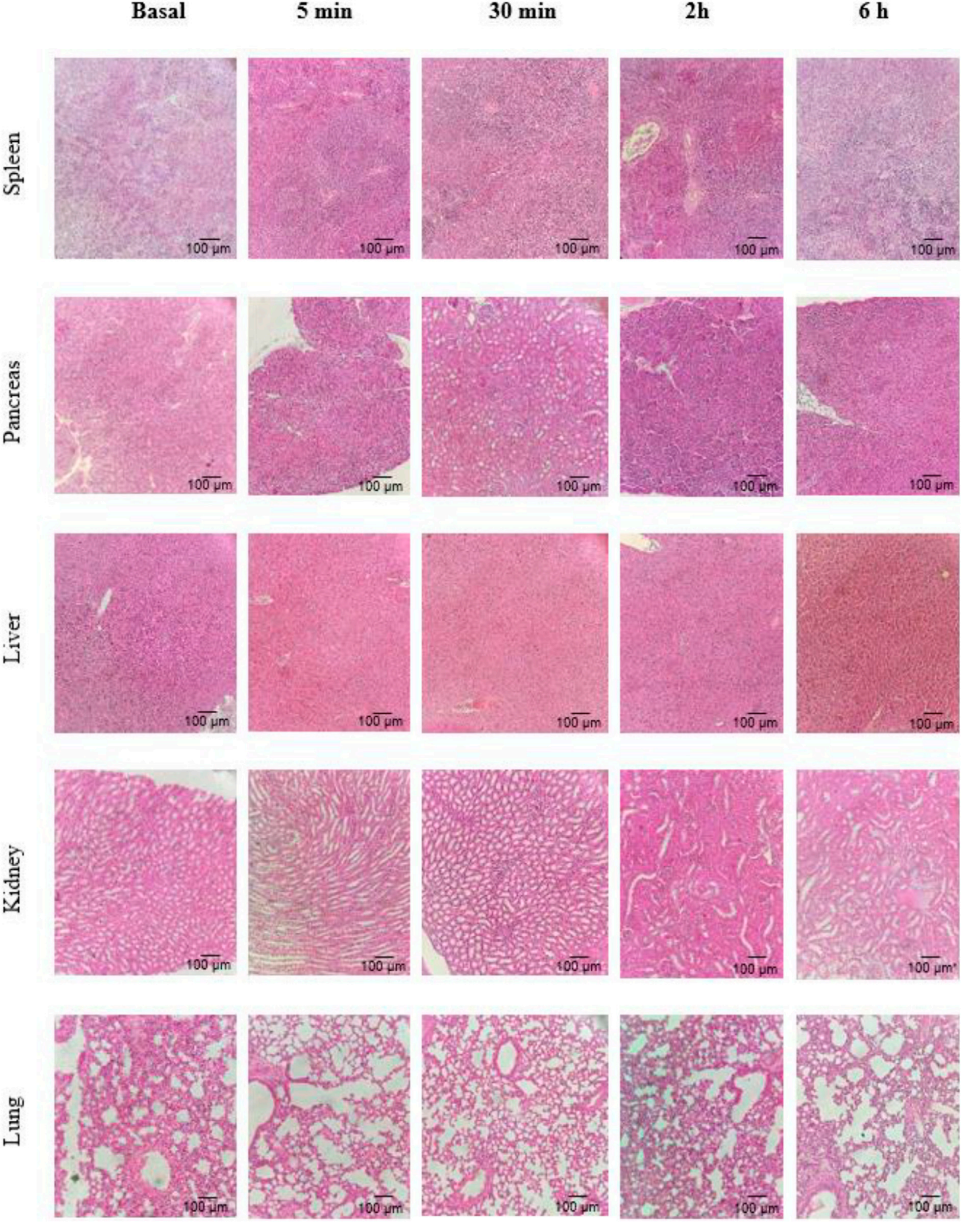
Representative histological sections of five organs collected in certain times after the injection of the peptide (*n* = 3).

After collection, the spleen, liver, pancreas, lungs, and kidneys were subjected to histological analysis. Figure 6 shows a representative image of *n* = 3 of each organ in a region randomly selected under a light microscope (×100), after 5 and 30 min and 2 and 6 h after peptide injection. No alterations were detected in any organ or at any time after the peptide injection, regarding cell/tissue morphology or the presence of an inflammatory infiltrate, similar to the same aspect as the basal group.

### 3.5 Oligomeric Aβ42

**FIGURE 7:**
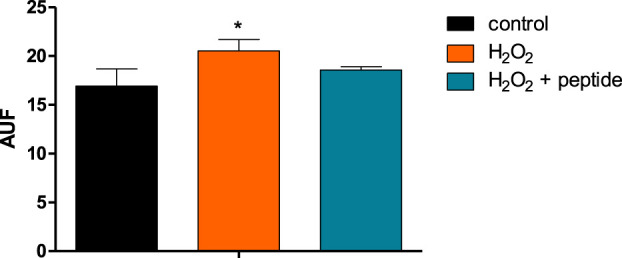
Peptide Aβ42 oligomerized and detected by fluorescence after labeling with thioflavin-T after activation of BACE-1 by H_2_O_2_, and the action of the peptide inhibits the enzyme and reduces the amyloid peptide. Data are present as the mean ± SD (*n* = 3) and * indicates *p* < 0.05 in a one-way ANOVA followed by Tukey’s post-test.

Differentiated neurons SH-SY5Y, treated with H_2_O_2_ for BACE-1 activation, could release oligomeric amyloid peptide 42 (Aβ42o), detected by thioflavin-T labeling, as shown in Figure 7, where the arbitrary units of fluorescence (AUF) were significantly increased with the treatment. With the incubation of the peptide, the Aβ42o levels were reduced, confirming and validating, in an *in vitro* assay, the activity of BACE-1 inhibition.

## 4 Discussion

BACE-1 is an important enzyme involved in APP processing and amyloid-peptide generation that contributes to the progression of neurodegenerative diseases (Zhao et al., 2020). Considering that BACE-1 is the key enzyme for the generation of *ß*-amyloid peptide 40 and 42, its inhibition is a relevant pharmacological target for disease treatment.

The first BACE-1 inhibitor developed was OM99-2, which is a peptidomimetic. Other molecules have been designed to increase potency with the development of inhibitors based on hydroxyethylene, hydroxyethylamine, carbamine, macrocycles, and non-peptidomimetics, such as acylguanidines, aminoimidazoles, aminohydantoin, and aminoquinazoline (Ghosh et al., 2012).

BACE-1 inhibitor peptides have also been isolated from marine animals. One example is a peptide generated from the hydrolyzed muscle of the gastropod *Aplysia kuroda*, a competitive inhibitor of the enzyme (Lee et al., 2019).

Here, we present a new peptide that is a competitive inhibitor of BACE-1. This peptide was isolated from *Merluccius productus* fish by Lee et al. (2019) and slightly modified by our group to be less charged and suitable to permeation through the blood–brain barrier. This peptide was chosen because it has a similar amino acid sequence and hydrophobicity pattern in peptides characterized by our group [(Banagouro et al., 2022) and data not shown], with BACE-1 inhibitory activity determined *in silico*.

The first analysis was performed to determine whether the new peptide maintained BACE-1 inhibitory activity. We first performed molecular docking analysis, which showed the binding of the peptide to the catalytic pocket of the enzyme. The first N-terminal residues were able to bind to BACE-1 at short distances, indicating stable binding. Moreover, the last residue (Asn) also bonded to the enzyme, as Lee et al. have already shown; the dipeptide Leu–Asn showed the most potent activity among other fragments derived from the original peptide, in addition to reducing the Aβ production in SH-SY5Y cells. Therefore, these two hydrophilic amino acids are important for maintaining the peptide structure and binding to the BACE-1 active site. Other amino acids have important functions in membrane permeation due to their hydrophobic features.

Although the peptide did not bind to the residues from the catalytic dyad (Asp32/Asp228), the molecule interacted to adjacent amino acids (Thr232, Asn233, and Ser229), placed exactly in the catalytic pocket, with hydrogen-bond distances. The nine-residue amino acids would compete with the peptide/protein substrate, impairing its positioning in the catalytic pocket, consequently inhibiting the activity of the BACE-1 (Rawlings et al., 2018).

The catalytic residues are placed at the center of the catalytic pocket, forming a hydrogen network for molecule binding. However, other amino acids are important for inhibitor stabilization, such as S2 and S4 subsite residues, which are mostly hydrophilic, and S1 and S3, which are hydrophobic. One example is the OM99-2 inhibitor, in which its P1 leucine and P3 valine fill the S1 and S3 hydrophobic sites and bind to Tyr71 and Phe108 from BACE-1 (Ghosh and Osswald, 2014). Similarly, our peptide inhibited BACE-1 by binding in other residues than the catalytic dyad.

Moreover, the peptide bound to Gln73, a residue from the BACE-1 flap. The flap from BACE-1 is the most flexible region from the structure that controls the substrate to reach the catalytic site by covering the active site. Thus, this flap has several multiple conformational states in different crystal structures, but it is closed when inhibitors are attached. The binding of Gln73 to our peptide in different flap conformations reinforced the BACE-1 inhibition profile.

Inhibitory compounds that bind to Gln73 also showed important BACE-1 inhibition, such as pyrazinecarboxamide, which had an IC50 < 1 nM. Moreover, this compound reduces Aβ levels in the rat brain (Wu et al., 2016).

Therefore, the positioning of the peptide in the catalytic pocket of BACE-1 and coordinating the closing of the flap characterized a competitive inhibition. These data were confirmed experimentally, and an observation of a competitive inhibitor agreed with the molecular docking analysis, in which the peptide was placed in the active site of the enzyme, including the flap backbone. Moreover, we determined the inhibitory constant (Ki), which was calculated to be 94 nM, indicating a good enzyme inhibition.

The BACE-1 inhibition could be confirmed by *in vitro* experiments, in which differentiated neurons were stimulated with H_2_O_2_ to activate BACE-1. Tan et al. (2013) demonstrated that oxidative stress increased BACE-1 protein levels and initiated the amyloidogenic pathway of APP, generating amyloid peptides. Our data agreed with this finding, where 100 µM H_2_O_2_ increased oligomerized Aβ42 levels, detected by a conventional approach of thioflavin-T labeling. We saw that when the peptide was present, these levels were reduced to the basal levels, confirming the peptide as the BACE-1 inhibitor in cells.

Moreover, it was verified that the peptide is not capable of being cleaved by plasma enzymes, as it does not have residues for such hydrolysis, which allows the administration of the peptides in a living organism, e.g., mice. Thus, we opted for an intravenous injection to avoid unspecific cleavage by stomachal and intestinal/pancreatic enzymes after an oral administration. The intravenous route is the first choice for protein and peptide drugs due to the possible enzymatic processing and, consequently, peptide loosing (Bruno et al., 2013). We could observe the intact mass of the complete peptide, confirming that there was no enzymatic cleavage, and no fragments were observed in mass spectrometry analysis (data not shown). Therefore, these data surpass the main disadvantage of a peptide drug, maintaining its advantages—high specificity and potency (Wang et al., 2022).

The peptide was found in the plasma at all analyzed times, with the highest concentration 5 min after the injection, and decreased over time, typical of an intravenous administration. We calculated the PK parameters using the compartmental analysis of the plasma after an intravenous bolus injection, and we could obtain the peptide concentration at time zero and values for distribution and elimination. The half-life was calculated at 0.7 h, similar to the value calculated by the computational model.

Due to the increased metabolic rates of mice, compared to human, the drug distribution and elimination profile is usually faster, but these data would help us to select an effective dose without causing any toxicity (Benet and Zia-Amirhosseini, 1995).

In pancreas, the peptide could not be found, but it was detected in other organs, showing that, after its entry into the systemic circulation, it was distributed. The distribution is not homogeneous due to the difference in blood perfusion, that is, the association with the tissues, pH, and permeability of the cell membrane.

In our study, it was possible to quantify the peptide at all times in spleen and liver (from 5 min to 6 h), with a peak at 30 min. The spleen is an organ of great importance for the immune system; it is highly irrigated, and a great exposure to external agents is expected, as we observed here (Cataldi et al., 2017). Nevertheless, the accumulation of the peptide indicates no toxicity for the organ. The liver is the main site of xenobiotic metabolism, where biotransformation reactions occur, generating more polar compounds to facilitate elimination from the body (Almazroo et al., 2017). The decrease over time indicates that there was no accumulation of the peptide in hepatocytes, which suggests not only the absence of toxicity but also the possible participation of the organ in the peptide metabolism.

The lack of toxicity could be confirmed by histological analysis, where the tissue was maintained intact after a single-dose administration of the peptide, and no inflammatory cells were observed. Thus, the biodistribution in these tissues was quite rapid, insufficient to cause any disturbance.

In kidneys, the peptide was observed after 30 min and increased after 6 h. The kidneys are mainly responsible for the elimination of peptides, but it also participates in metabolism, since it has peptidases in the proximal tubule. The elimination of small peptides, such as the peptide presented here, can occur freely by the kidneys, as they can enter and filtered by the glomerulus (Brater, 2002). This indicates that the urinary tract is the main route of elimination of the peptide in its intact primary structure.

The peptide was also detected in the lungs, from 5 min to 6 h. It is not common for a peptide to permeate lung cells due to its size, but its hydrophobicity nature may have contributed to pass membranes, as lipophilic molecules/drugs can easily permeate the airway epithelium via the passive transport (Matthews et al., 2020). Similarly, a small protein with the antitumor effect, Amblyomin-X, was found in the lungs in high concentration 10 min after an intravenous administration, and our findings show that no toxicity was related to this protein (Boufleur et al., 2019). Nevertheless, no clinical signals of toxicity were observed after a single dose of the peptide shown here.

Interestingly, the peptide was found in the brain, confirming the prediction of the hydrophobicity, which indicates the membrane permeation, including the brain–blood barrier. The hydrophobic nature of the peptide allowed its fast distribution, as it probably passed membranes easily, besides the presence of hydrophilic amino acids in the N- and C-terminal, which facilitates binding to the aqueous solution and polar portion of the membrane.

The presence of the peptide in the brain is essential, considering that the target is BACE-1 from neurons to treat a neurodegenerative disease. Moreover, the peptide could be found up to 6 h in the tissue, showing an effective time for the molecular-target interaction. The *in vivo* efficacy of the peptide will be further studied.

## 5 Conclusion

In conclusion, we optimized a peptide using *in silico* tools to obtain a potent and competitive inhibitor of BACE-1, which is able to distribute and permeate the brain–blood barrier without causing any toxicity in mice and is useful for the treatment of amyloid-linked neurodegenerative diseases.

## Data Availability

The datasets generated for this study can be found in www.inovamol.com.br.
